# Evaluation of the osseointegration of dental implants coated with calcium carbonate: an animal study

**DOI:** 10.1038/ijos.2017.13

**Published:** 2017-04-28

**Authors:** Yi Liu, Yi Zhou, Tao Jiang, You-De Liang, Zhen Zhang, Yi-Ning Wang

**Affiliations:** 1 Department of Stomatology, Huangshi Central Hospital, Affiliated Hopsital of Hubei Polytechnic University, Edong Healthcare Group, Huangshi, China; 2 The State Key Laboratory Breeding Base of Basic Science of Stomatology (Hubei-MOST) & Key Laboratory of Oral Biomedicine, Ministry of Education, School and Hospital of Stomatology, Wuhan University, Wuhan, China

**Keywords:** calcium carbonate, histomorphometry, *in vivo*, osseointegration, titanium

## Abstract

In an attempt to overcome the limitations of titanium in dental and orthopaedic clinical applications, a new method has been developed to prepare calcium carbonate coatings on sandblasted and acid-etched (SA) titanium implants. The purpose of this study was to investigate the effect of calcium carbonate-SA (CC-SA) implants on osseointegration *in vivo*. The surfaces of SA and CC-SA implants were characterised for surface morphology and surface chemistry. Subsequently, these two kinds of implants were implanted in the femoral condyles of rabbits. The implants were retrieved and prepared for histological and histomorphometric evaluation 1, 2, 4, 8 and 12 weeks after implantation. Significantly higher values of bone-to-implant contact of the entire implant except the gap area (BIC_ALL) and the bone-to-implant contact of the gap area (BIC_GAP) were found in animals with the CC-SA implants than in those with the SA implants at 4 weeks. Higher values of total gap bone were found in those with the CC-SA implants than in those with the SA implants at 1, 2 and 4 weeks. In conclusion, the current findings demonstrate that the calcium carbonate coating can improve and accelerate the early ingrowth of bone and osseointegration at the early healing phase. This may reduce clinical healing times and thus improve implant success rates.

## Introduction

Titanium is one of the most commonly used materials for dental and orthopaedic implants on account of its high biocompatibility, chemical stability and excellent mechanical properties.^1, 2^ However, the oxide film that spontaneously forms on titanium when it is exposed to oxygen makes it bioinert.^3^ Modifications of metal surfaces are often employed as a means of controlling the implant–tissue interaction and osseointergration.^4^

Applying bioactive coatings on titanium implants is one of the most important ways to improve surface properties,^5^ so that the mechanical strength of the titanium implants and the bioactivity of the coatings are well combined. HA-coated surfaces achieve a very intimate bone-to-implant contact and have been claimed to reduce the healing period. However, a retrospective long-term clinical study on 313 HA-coated oral implants reported that the cumulative survival rate decreased to 77.8% after 8 years for HA-coated implants, compared with 92.7% for titanium plasma-sprayed implants.^6^ Another clinical report also showed that the cumulative success rates of HA-coated implants after 5 and 10 years were 89.9% and 54%, respectively.^7^ The long-term effects of HA-coated implants may be limited by vulnerable interface attachment between the HA coating and the implant metal body,^8^ surface resorption-induced foreign body reactions^9^ and relatively low shear and fatigue strengths.^10^

Therefore, the idea of a resorbable coatings was developed based on the thought that an optimal coating should completely disappear before completion of the bone-healing process^11^ and the formation of the bone–titanium contact. Oh *et al.*^12^ found that thinner coatings have been shown to be as effective as thicker coatings with respect to enhancement of the early bone response. Moreover, degradation of these coatings can even be associated with increased osteoconductivity and enhanced bone–implant contact (BIC).^13^

Both calcium phosphate and calcium carbonate are resorbable *in vivo*. Biomimetic calcium phosphate is regarded as an ideal coating material; studies have shown that it can promote bone formation.^14, 15^ Like calcium phosphate, calcium carbonate has been widely used as a bone substitute for decades.^16, 17^ Compared to calcium phosphate, calcium carbonate has a higher solubility.^18^ Therefore, some research has investigated the combination of calcium phosphate and calcium carbonate as a biphasic biomaterial.^19^

Calcium carbonate has been shown to be a biocompatible and osteoconductive material in the form of either aragonite or calcite.^20, 21^ Most importantly, calcium carbonate is degradable *in vivo*, making it a favourable candidate for implant coating, but no report on the use of this material has been available because of the technological difficulty of applying it as a coating. Recently, however, a method was developed to deposit calcium carbonate coatings on silicon wafers.^22^ Inspired by this, we used the new method to apply a calcium carbonate coating to sandblasted and acid-etched titanium (SATi) implants, a clinically successful implant. Our previous research has shown that calcium carbonate coating may yield a better biological outcome than titanium implants *in vitro* because it may induce differentiation towards an osteoblastic phenotype and therefore enhance the osteointegration process, especially in the early stage.^23^ The aim of the present study was to investigate the characteristics and *in vivo* bone formation of calcium carbonate-coated implants.

## Materials and methods

### Implant preparation

In total, 36 commercial pure titanium implants were machined for this *in vivo* study. The dumbbell-shaped implant was 3.3 mm in diameter and 8 mm in length. A gap was made in the middle of the implant, 2 mm long and 0.2 mm wide. The gap enabled investigation of the new bone formation, which is very difficult to assess on an implant surface that is in contact with the bone of a drilled hole. All implants were sandblasted with 0.25–0.50 mm corundum grit at 5 bars for 1 min. Subsequently, the implants were acid-etched in hydrochloric acid/sulfuric acid (1:1) at 65 °C for 30 min. After the above treatments, the implants were ultrasonically cleaned for 15 min in acetone, ethanol (70%) and deionised water, and finally dried at room temperature. Eighteen implants were coated with calcium carbonate; the other 18 implants served as the control.

### Calcium carbonate coating

**Figure 1:**
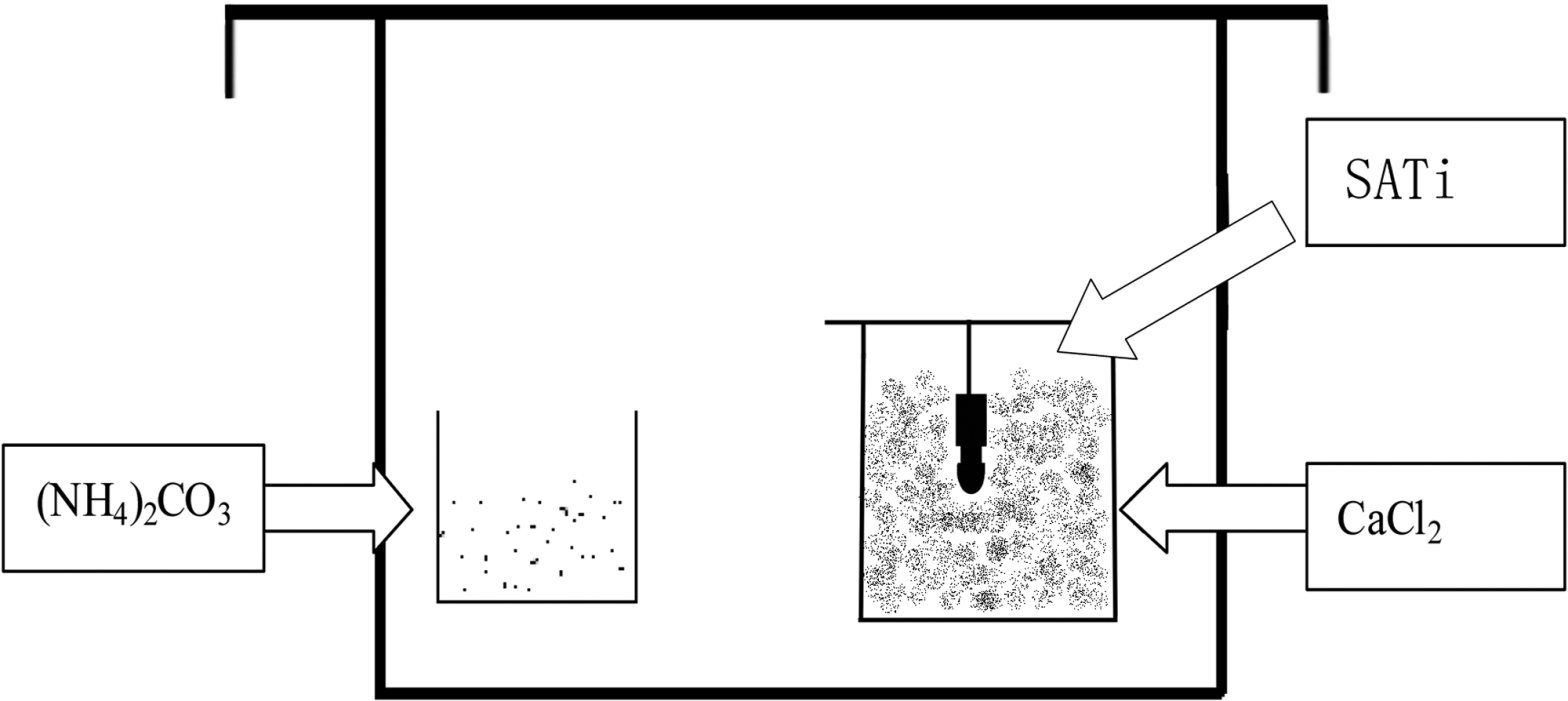
**Illustration of the experimental setup for preparation of the calcium carbonate coating on the SA implant surface**. Two vials—one containing a 20 mmol·L^−1^ CaCl_2_ solution, the other containing ammonium carbonate powder—were placed in a desiccator. SATi implants were placed vertically in the CaCl_2_ solution. CaCl_2_, calcium chloride; SATi, sandblasted and acid-etched titanium.

The experimental setup for preparing the CaCO_3_ films is illustrated in Figure 1. Two vials—one containing a 20 mmol·L^−1^ calcium chloride (CaCl_2_) solution, the other containing ammonium carbonate powder—were placed in a desiccator. SATi implants were placed vertically in the CaCl_2_ solution. CaCO_3_ films were then deposited on the SATi substrates *via* slow diffusion of CO_2_ produced by decomposition of ammonium carbonate at room temperature for 4 h. The samples were rinsed with deionised water for 1 min after deposition and then air-dried overnight. Through this process, calcium carbonate-SATi (CC-SATi) implants were produced. All implants were sterilised by autoclaving before surgery (for 15 min at 121 °C).

### Surface characteristics of implants

Two test and two control implants were used for surficial analysis.

A field-emission scanning electron microscope (FESEM, JEOL JSM-6700F; JEOL, Tokyo, Japan) was used to investigate the morphology and microstructure of the surfaces at an operating voltage of 5 kV. During the Fourier transform infrared (FTIR) test, the coating was scraped from CC-SATi surfaces and collected. The FTIR spectra for the coating were measured by FTIR spectroscopy (Nicolet 170SX; Madison, WI, USA). FTIR bands were identified *via* comparison with the literature values.

### Surgical procedure

The study protocol was approved by the Ethics Committee for Animal Research, Wuhan University, China. A total of 16 adult male New Zealand white rabbits, 8–12 months of age and weighing between 2.0 and 2.5 kg, were used in the study. The animals were housed in single boxes under the same environmental conditions (22 °C±1 °C, 55%±5% relative humidity), and they were fed a standard diet and filtered tap water. For surgery, 16 rabbits were anaesthetised with intramuscular injections of SMX compound anaesthesia (Institute of Veterinary, University of PLA, Changchun, China) at a dose of 0.3 mL·kg^−1^ body weight. SMX compound anaesthesia consisted of exylazole, ethylene diamine tetraacetic acid, dihydroetorphine hydrochloride and haloperidol. Each rabbit received one SA implant and one CC-SA implant at the same position in the femoral condyles close to the knee joint. All operations were performed by the same experienced implantologist. The antibiotic (ampicillin sodium; 25 mg·kg^−1^ intramuscularly daily) was administered for 5 days.

One rabbit was complicated by infection and was not included in any of the experimental or control groups. The remaining 15 rabbits survived the treatment and were available for evaluation.

At each time point (1, 2, 4, 8 and 12 weeks post surgery), three animals were killed *via* intravenous injections of an overdose of SMX compound anaesthesia. The clinical examination performed after death showed that all the implants were clinically stable.

### Histological and histomorphometric procedures

Femoral condyles with implants, processed for ground sectioning, were immediately fixed with 4% neutral-buffered paraformaldehyde, dehydrated in an ascending series of ethanol concentration, placed in xylene and subsequently embedded in methylmethacrylate in vacuum. Non-decalcified sections were prepared using a slow-speed saw with coolant (Leica SP1600; Leica Spa, Milan, Italy). These sections were made in a transversal direction perpendicular to the axis of the implant. Sections were then ground to a final thickness of 20 μm and stained with basic fuchsin and methylene blue.

Three sections were taken from each specimen for histological and histomorphometrical evaluation. An independent examiner performed histological analysis using a microscope equipped with an imaging system (Q-500 MCA; Leica, Wetzlar, Germany). Photographs of histomorphometric analysis were acquired using a computer-based National Institutes of Health image analysis system.

The histomorphometrical analysis consisted of the following measurements.

1. The amount of bone contact at the interface (BIC): the percentage of implant length showing a direct bone-to-implant contact without any intervening soft tissue layer. The parameter was further differentiated into the percentage of bone-to-implant contact of the entire implant except the gap area (BIC_ALL) and the percentage of bone-to-implant contact of the gap area (BIC_GAP). The gap area, which was in the middle of the implant, was 2 mm long and 0.2 mm wide.
2. Total gap bone (TGB): the amount of bone inside the gap between the implant and the cancellous bone.
3. Bone volume/tissue volume (BT05): percent bone area at a distance of 0.5 mm from the implant surface except the gap area.

### Statistical analysis

Statistical analysis was performed using SPSS 10.0 for Windows (Seattle, WA, USA). Data are reported as the mean±standard deviation at a significance level of *P*<0.05. Paired *t*-tests were used for the comparison of parameters between the SA and CC-SA groups at the same healing time.

## Results

### Surface characterisation

**Figure 2:**
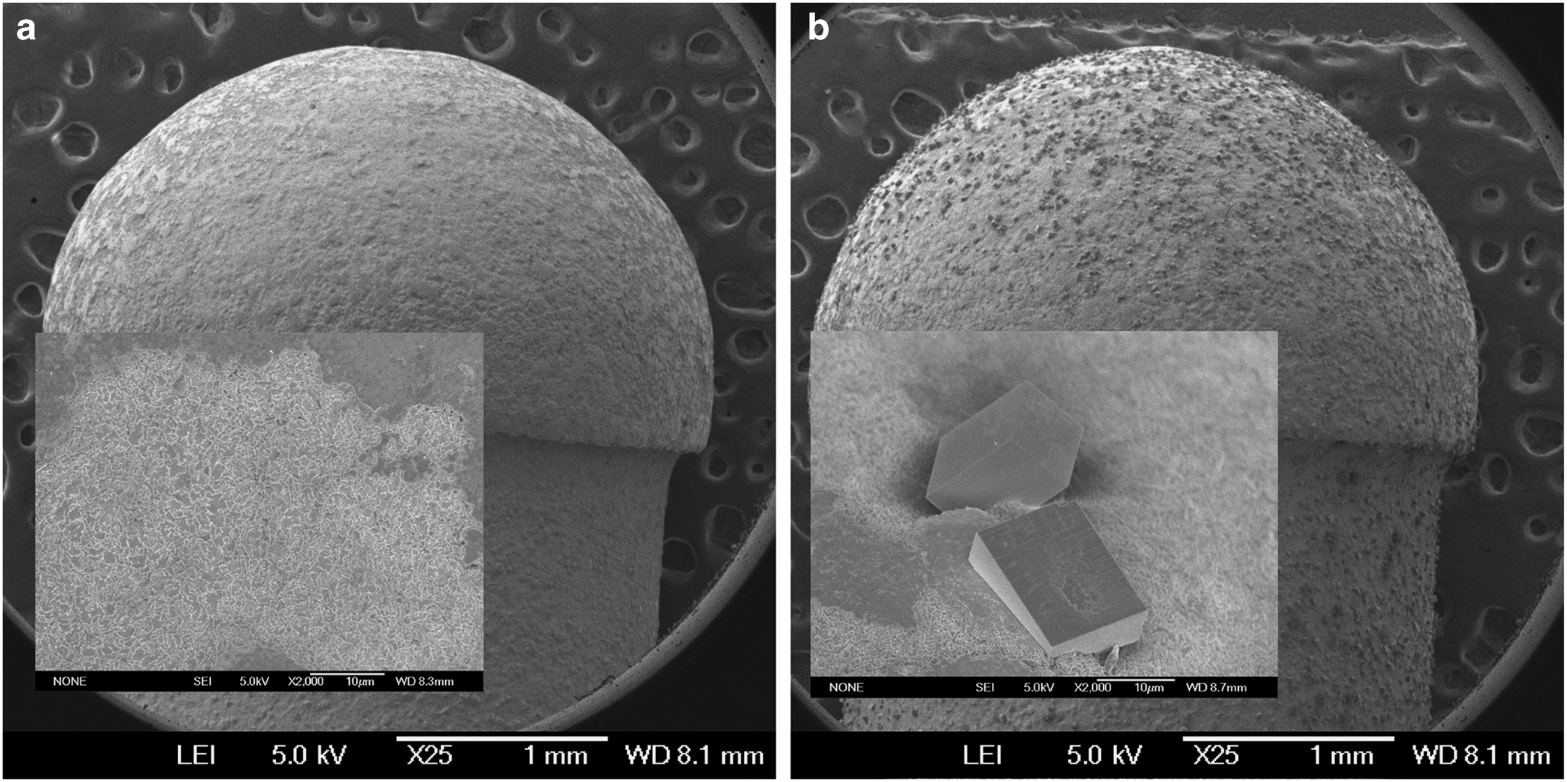
**Field-emission scanning electron microscope micrographs showing the surface topography of the tested titanium implants**. (**a**) SATi; (**b**) CC-SATi. CC-SATi, calcium carbonate-SATi; SATi, sandblasted and acid-etched titanium.

FESEM micrographs of the SATi and CC-SATi surfaces are shown in Figure 2. SATi surfaces contained pits and craters (Figure 2a). The pits were 0.5–3 μm in diameter and appeared to coalesce to form large craters. Crystals with a rhombohedral and needle-like shape were observed on the CC-SATi surfaces (Figure 2b). They formed discontinuous coatings on the substrate. Some crystals partly contacted each other, and some were separated from the neighbouring crystals. Gaps between the separated crystals ranged from several μm to 20 μm.

**Figure 3:**
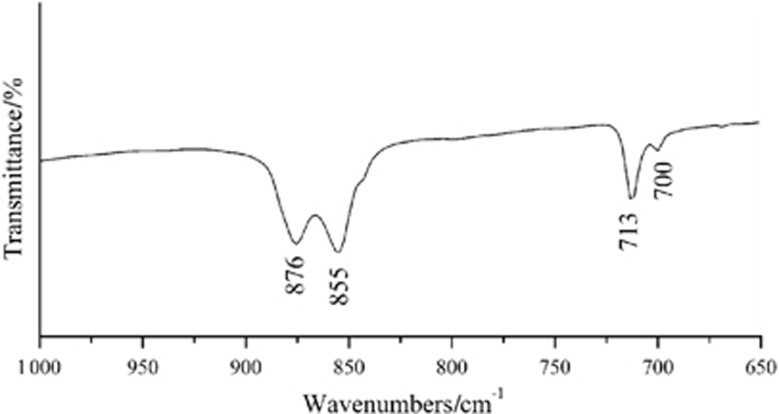
**Fourier transform infrared spectra of CaCO_3_ crystals deposited on the sandblasted and acid-etched titanium surface.**

The FTIR spectra of crystals deposited on CC-SATi are presented in Figure 3. The precipitates were mainly a mixture of calcite and aragonite. Two sharp absorption bands were found at 876 and 713 cm^−1^, which was the characteristic frequency of well-formed calcite. The spectra also displayed the characteristic absorption peaks of aragonite at 855, 713 and 700 cm^−1^.

### Experimental animals

During the experiment, all 15 rabbits remained in good health and did not show any wound complications. A total of 30 implants were retrieved after the animals were killed. The implant sites showed no sign of inflammation, and the implants were clinically stable at the time of harvest.

### Histology results

**Figure 4:**
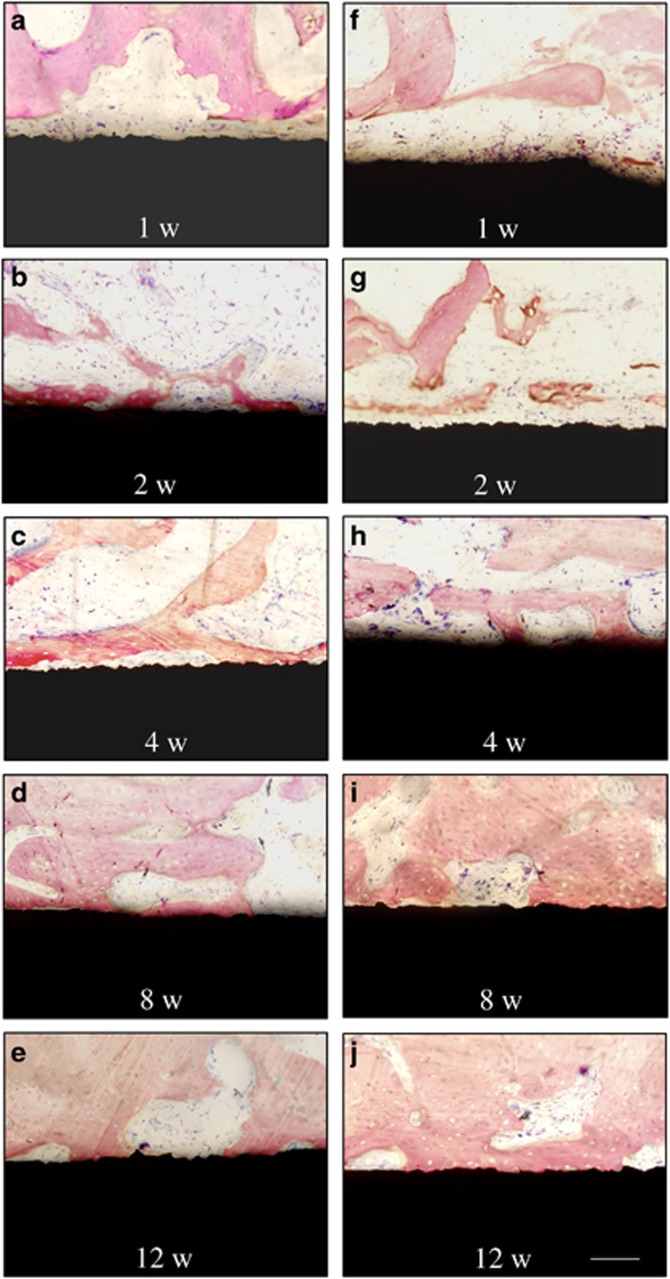
**Light micrographs of bone–implant interfaces.** SA implants implanted in rabbits at 1 (**a**), 2 (**b**), 4 (**c**), 8 (**d**), 12 weeks (**e**) and CC-SA implants implanted in rabbits at 1 (**f**), 2 (**g**), 4 (**h**), 8 (**i**), 12 weeks (**j**). Basic fuchsin and methylene blue staining, × 20. Scale bar=100 μm. CC-SA, calcium carbonate-SA; SA, sandblasted and acid-etched.

**Figure 5:**
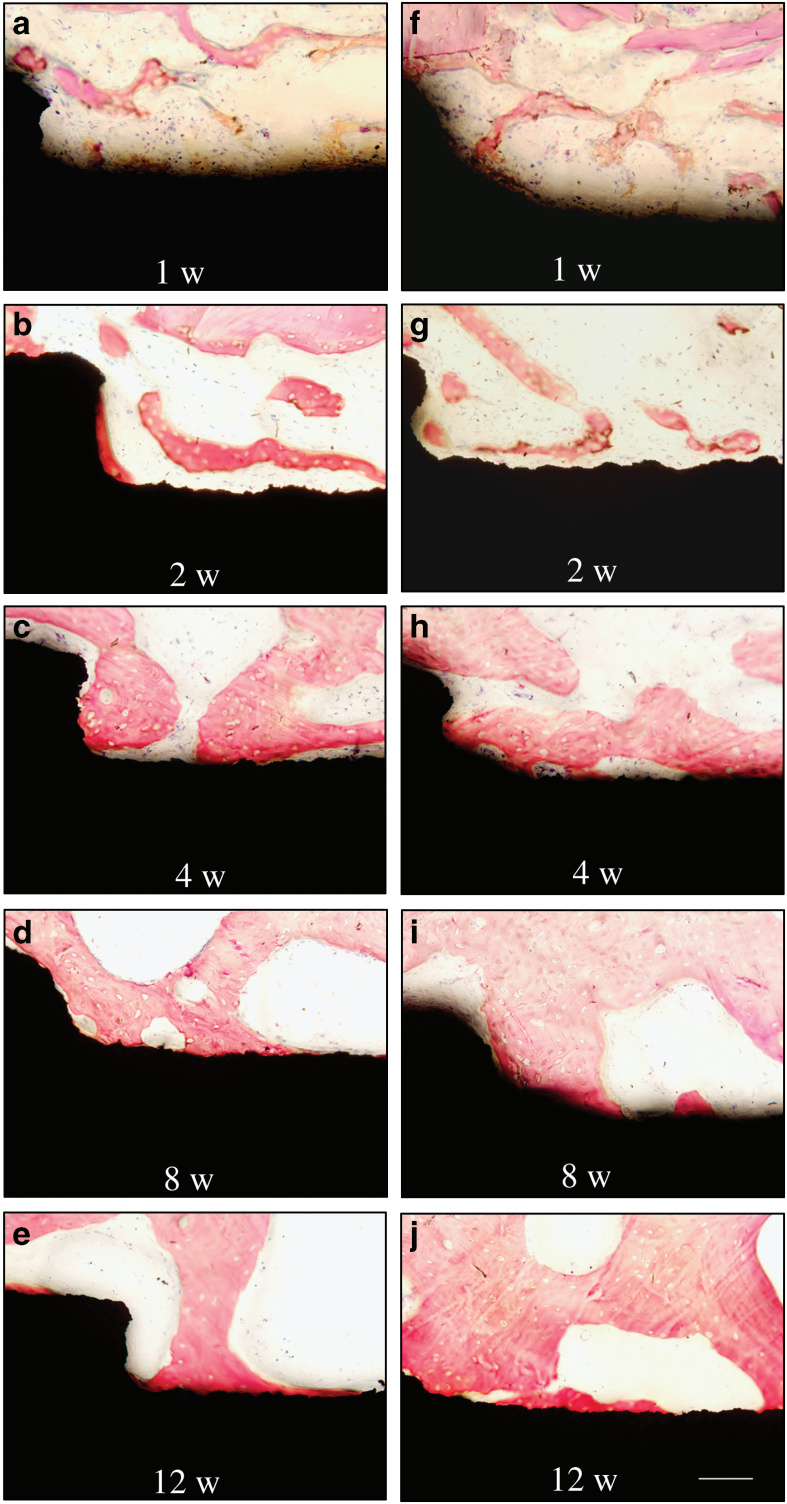
**Light micrographs of the gaps.** SA implants implanted in rabbits at 1 (**a**), 2 (**b**), 4 (**c**), 8 (**d**), 12 weeks (**e**) and CC-SA implants implanted in rabbits at 1 (**f**), 2 (**g**), 4 (**h**), 8 (**i**), 12 weeks (**j**). Basic fuchsin and methylene blue staining, × 20. Scale bar=100 μm. CC-SA, calcium carbonate-SA; SA, sandblasted and acid-etched.

Light micrographs of the bone–implant interface are shown in Figures 4 and 5. At 1 week, there was no obvious difference between the CC-SA and SA implant at high magnification. A gap between the old bone and the implant surface was observed in most areas. At 2 weeks, new bone formation was present around all implants in the cancellous bone. In both groups, many cubical osteoblasts were present and contacted the bone matrix near the implant surface. At 4 weeks, bone modelling was observed for the two kinds of implants, where lamellar bone was observed surrounding an abundant vascular structure. There seemed to be more osteoblasts in the area near the surface of the CC-SA implant than near the SA implant. At 8 weeks, Haversian systems were observed for both groups. At 12 weeks, mature bone with well-mineralised osteoid was present in both groups. Flat lining cells were observed on the surface of trabeculae.

### Histomorphometric results

**Figure 6:**
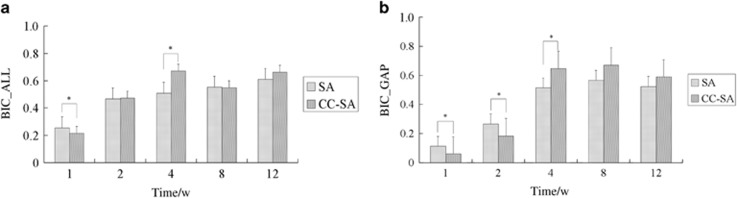
**Bone-to-implant contact, expressed as the percentage of implant length showing a direct bone-to-implant contact without any intervening soft tissue layer.** The parameter was further differentiated into (**a**) the percentage of bone-to-implant contact of the entire implant except the gap area (BIC_ALL) and (**b**) the percentage of bone-to-implant contact of the gap area (BIC_GAP). Error bars represent means±standard deviation for *n*=6. *P<0.05, **P<0.01. CC-SA, calcium carbonate-SA; BIC, bone-to-implant contact; SA, sandblasted and acid-etched.

All bone apposition data for the two kinds of materials are shown in Figure 6. Significantly higher values of BIC_ALL were found in the SA implants than in the CC-SA implants at 1 week, but at 4 weeks they were higher in the CC-SA implants than in the SA implants (Figure 6a). At 1 and 2 weeks, significantly higher values of BIC_GAP were found in the SA implants than in the CC-SA implants (Figure 6b). In comparison with the values for the SA implants, higher values of BIC_GAP were found in the CC-SA group at 4 weeks. The BIC_ALL and BIC_GAP values showed no statistically significant difference at 8 and 12 weeks.

**Figure 7:**
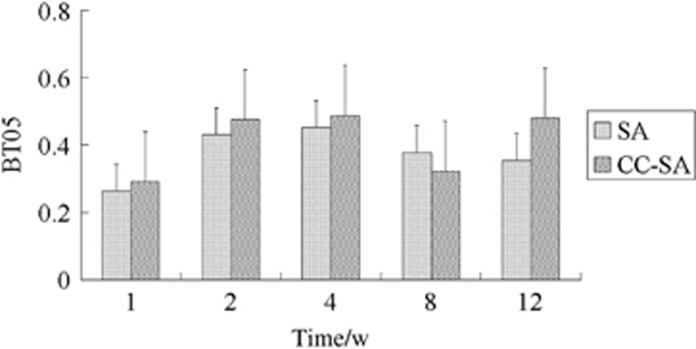
**Bar graph showing the percent bone area at a distance of 0.5 mm from the implant surface except the gap area (BT05).** Significant differences were not found between CC-SA and SA implants. Error bars represent means±standard deviation for *n*=6. CC-SA, calcium carbonate-SA; SA, sandblasted and acid-etched.

**Figure 8:**
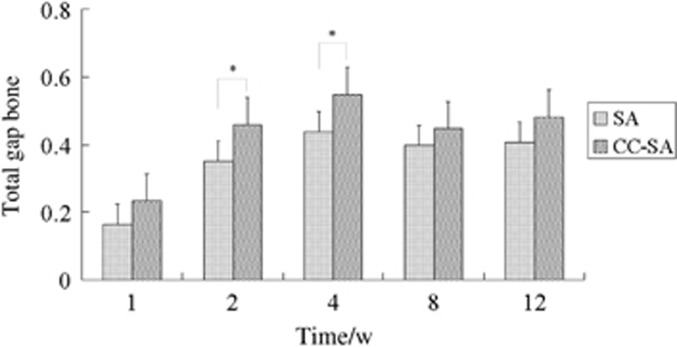
**Bar graph showing the amount of bone inside the gap between the implant and the cancellous bone.** The TGB in the CC-SA implants was significantly higher than in the SA implants at 1, 2 and 4 weeks. Error bars represent means±standard deviations for *n*=6. *P<0.05, **P<0.01. CC-SA, calcium carbonate-SA; SA, sandblasted and acid-etched; TGB, total gap bone.

The data for the bone volume/tissue volume near the implant surface (BT05) are shown in Figure 7. There were no significant differences between CC-SA and SA surfaces at any of the time points. A higher value of TGB, an index of new bone formation, was found in the CC-SA implants than in the SA implants at 1, 2 and 4 weeks but not at 8 and 12 weeks. (Figure 8).

## Discussion

Our idea for developing an implant coating was to construct a resorbable layer that accelerates new bone formation at the early phase of osseointegration and disappears before the bone makes contact with the titanium. This idea is based on the clinical observation that the stability of implants is significantly decreased at 3–4 weeks post surgery compared to readings obtained at surgery, but subsequently increases.^24, 25^ Secure primary stabilisation followed by robust osseointegration are of critical importance for the clinical result and the long-term stability of the implant.^26^ The aim of the calcium carbonate coating is to minimise the period of reduced stability and promote osseointegration at the early healing stage.

We found that calcium carbonate-coated surfaces significantly enhanced bone-to-implant contact (BIC_ALL) at 4 weeks. Furthermore, the new bone formation in the gap (BIC_GAP) was also higher than in the SA implants at 2 and 4 weeks, which is consistent with previous reports.^16, 17, 20, 21^ The results suggest that the enhanced bone-to-implant contact may have been due to superior new bone formation in the calcium carbonate group. The positive effect of the calcium carbonate coating may be based on the stimulation of integrin-mediated osteoblast response by calcium ions through the enhanced ligand binding of the integrin receptor.^27, 28^ Physical effects such as changes in surface energy due to implant coating and enlargement of the surface area may also play a role.^29^ Surface roughnesses and the contact angles of CC-SATi and SATi surfaces were measured in our *in vitro* research. We observed that calcium carbonate crystals, especially rhombohedral ones, made the CC-SATi surfaces rougher than SATi surfaces. However, no significant differences in their contact angles were observed between CC-SATi and SATi.^23^ High surface energy, considered an important variable, was found to be insufficient to cause a marked increase in osteoblast responses to Ti substrates with low surface roughness. In contrast, when substrates with complex micron-scale and submicron-scale roughness are fabricated to retain the high surface energy of uncontaminated TiO2, the cells exhibit synergistic enhancement of their response compared to the surface topography alone.^30^

In this study, calcium carbonate coatings were deposited on SA implants using a simple chemical treatment. The SA implants performed well owing to their original titanium properties.^31^ The microporous surface of titanium shows a large peak-to-valley pore height and is thought to increase cell attachment to promote osteoblast differentiation.^32^ Once a CC-SA implant improves peri-implant bone regeneration beyond the effect of the SA implant alone, it can be assumed that a CC-SA implant is an optional material for clinical use.

However, the opposite results were found for BIC_ALL at 1 week and BIC_GAP at 1 and 2 weeks. An explanation for this might be that at an early healing time, some bone directly adjacent to the calcium carbonate was not included in the BIC.

It should be noted that the bond strength of the coating could not be appropriately evaluated using conventional methods such as a shear, tensile or scratch test because the coating was discontinuous. In addition, the thickness is hard to measure. During the FTIR test, we found that it was difficult to scrape the coating from the CC-SATi surfaces. Moreover, in this experiment, the calcium carbonate-coated implants were found to significantly enhance bone-to-implant contact (BIC_ALL) at 4 weeks. The new bone formation in the gap (BIC_GAP) was also higher than in the SA implants at 2 and 4 weeks. This indicated that the calcium carbonate coating could withstand peeling during the implant procedure. The bond strength is sufficient for clinical use.

Implants observed at 8 and 12 weeks did not show any significant difference in BIC_ALL and BIC_GAP. This may indicate that the coating was resorbed and the enhancing effect of the calcium carbonate-coated implants as compared with the SA implants was limited to early stages of peri-implant bone formation.

In previous studies, Hernández-Hernández *et al.*^33^ prepared calcium carbonate using a vapour-diffusion technique.^33^ Isfisco *et al.*^34^ prepared calcium phosphate nanoparticles using the same technique. Further study is needed to determine whether this technique can be used to deposit calcium phosphate on SA implants and to assess the biological performances of these two kinds of implants.

The FESEM images showed that the titanium surface was only partially covered by the calcium carbonate crystals using the present coating technique. Therefore, our method needs to be improved to achieve an evenly coated calcium carbonate layer. Another limitation of this pilot study was the small number of animals.

## Conclusions

Our results confirm that CC-SA and SA implants show good biocompatibility and osteoconductive properties after a 12-week healing period. A calcium carbonate coating can improve and accelerate the early ingrowth of bone and osseointegration. This may reduce clinical healing times and thus improve implant success rates. Furthermore, the technique used here is a promising new method to deposit CaCO_3_ coatings onto titanium substrates and warrants further investigation.
